# Overlapping functions of the starch synthases SSII and SSIII in amylopectin biosynthesis in *Arabidopsis*

**DOI:** 10.1186/1471-2229-8-96

**Published:** 2008-09-23

**Authors:** Xiaoli Zhang, Nicolas Szydlowski, David Delvallé, Christophe D'Hulst, Martha G James, Alan M Myers

**Affiliations:** 1 Department of Biochemistry, Biophysics and Molecular Biology, Iowa State University, Ames, Iowa, USA; 2 The Ohio State University, Center for Biostatistics, M200 Starling Loving Hall, 320 W. 10th Avenue, Columbus, OH 43210, USA; 3 Unité de Glycobiologie Structurale et Fonctionnelle, UMR8576 du CNRS, IFR 147, Bâtiment C9, Université des Sciences et Technologies de Lille, 59655 Villeneuve d'Ascq Cedex, France

## Abstract

**Background:**

The biochemical mechanisms that determine the molecular architecture of amylopectin are central in plant biology because they allow long-term storage of reduced carbon. Amylopectin structure imparts the ability to form semi-crystalline starch granules, which in turn provides its glucose storage function. The enzymatic steps of amylopectin biosynthesis resemble those of the soluble polymer glycogen, however, the reasons for amylopectin's architectural distinctions are not clearly understood. The multiplicity of starch biosynthetic enzymes conserved in plants likely is involved. For example, amylopectin chain elongation in plants involves five conserved classes of starch synthase (SS), whereas glycogen biosynthesis typically requires only one class of glycogen synthase.

**Results:**

Null mutations were characterized in *AtSS2*, which codes for SSII, and mutant lines were compared to lines lacking SSIII and to an *Atss2*, *Atss3 *double mutant. Loss of SSII did not affect growth rate or starch quantity, but caused increased amylose/amylopectin ratio, increased total amylose, and deficiency in amylopectin chains with degree of polymerization (DP) 12 to DP28. In contrast, loss of both SSII and SSIII caused slower plant growth and dramatically reduced starch content. Extreme deficiency in DP12 to DP28 chains occurred in the double mutant, far more severe than the summed changes in SSII- or SSIII-deficient plants lacking only one of the two enzymes.

**Conclusion:**

SSII and SSIII have partially redundant functions in determination of amylopectin structure, and these roles cannot be substituted by any other conserved SS, specifically SSI, GBSSI, or SSIV. Even though SSIII is not required for the normal abundance of glucan chains of DP12 to DP18, the enzyme clearly is capable of functioning in production such chains. The role of SSIII in producing these chains cannot be detected simply by analysis of an individual mutation. Competition between different SSs for binding to substrate could in part explain the specific distribution of glucan chains within amylopectin.

## Background

Insoluble starch granules function as a central component of plant metabolism to store reduced carbon produced during photosynthesis. Starch is made up of two types of glucan homopolymer, amylose and amylopectin [1]. Amylose molecules typically comprise several thousand glucose units joined by α-(1→4) glycoside bonds, with a low frequency of branch points provided by α-(1→6) glycoside bonds. Amylopectin has a degree of polymerization (DP) on the order of 10^4 ^– 10^5 ^glucose units per molecule, and contains a relatively high frequency of branch linkages, approximately 5%. A specific distribution of α-(1→4)-linked glucan chain lengths, together with clustered positioning of α-(1→6) branch linkages, provides structure to amylopectin that allows crystallization and formation of insoluble starch granules [2-4]. The ability to crystallize in turn provides the functionality of starch because very large numbers of glucose units can be stored as an energy source to be used later when photosynthesis is not operative. Thus, an important objective towards the aim of fully understanding plant physiology is to determine how each of the enzymes involved in starch biosynthesis functions to produce a polymer with a crystallization-competent architecture.

Starch is produced by the coordinated actions of the following enzymes: 1) ADPGlc pyrophosphorylase (ADPGPP), which provides the nucleotide sugar donor ADPGlc; 2) starch synthase (SS), which catalyzes the transfer of a glucosyl unit from ADPGlc to a growing polymer chain through an α-(1 → 4) glycoside bond; 3) starch branching enzyme (SBE), which cleaves an internal α-(1 → 4) linkage and transfers the released linear chain to a C-6 hydroxyl, thus forming a new α-(1 → 6) branch point; 4) starch debranching enzyme (DBE), which selectively hydrolyzes α-(1 → 6) linkages and has been proposed to provide an editing function in selection of branch points [5-8]. Most organisms outside the plant kingdom possess a single class of glycogen synthase (GS), in some instances comprising two closely related isoforms, which catalyzes the same glycosyl transferase reaction as the plant SSs. In contrast, a highly conserved feature of starch biosynthesis in diverse plant species is the presence of five distinct classes of SS [9,10], again often with multiple isoforms in each class. Each SS class is found in unicellular green algae and thus likely was established prior to the evolution of land plants [11]. Such conservation suggests that each SS is functionally significant for synthesis of granular starch as opposed to water-soluble glycogen, presumably through its influence on the architectural structure of amylopectin. Some functions of SSs with regard to amylopectin architecture are relatively well understood, however, for the most part their functional relationships to each other remain to be determined.

One SS is nearly exclusively granule-bound (GBSS), whereas the other four classes are distributed between granules and stroma (SSI, SSII) or are located nearly entirely in the stroma (SSIII, SSIV) [12-18]. Genetic evidence indicates that all five SSs have specific functions that cannot be provided by any other SS class. For example, elimination of GBSS by mutation or antisense gene expression conditions loss of the amylose component of starch granules without major effects on amylopectin [19,20]. Thus, it appears that GBSS is specifically involved in amylose biosynthesis and that no other SS can provide this function. SSII deficiency in numerous species results in increased frequency of short glucan chains (DP6 to DP11) within amylopectin, decreased abundance of DP12 to DP25 chains, and in most instances an elevated amylose to amylopectin ratio [18,21-27]. A possible explanation for these results is that SSII catalyzes formation of chains of DP12 – DP25, and that the other SS isoforms cannot fulfil this function. The uniformity of the SSII-mutant phenotype also indicates that the physiological function of SSII is conserved across plant species, in addition to the paralogous amino acid sequences.

*Arabidopsis *is useful as a model organism for study of SS function, as well as starch biosynthesis in general, owing to genome-wide reverse genetic resources that can provide null mutations in all of the individual enzyme classes. Using such tools the effects of eliminating either SSI, SSIII or SSIV on *Arabidopsis *leaf starch have been determined. Mutations that eliminate SSI cause decreased frequency of the shortest linear chains within amylopectin, of DP6 to DP12 [28]. Loss of SSIII causes increased frequency of long glucan chains greater than DP60 but has relatively little effect on the abundance of any chains less than DP50 [29]. SSIV deficiency did not cause any noticeable changes in amylopectin structure, however, this enzyme has a function in initiation of starch granule formation [30].

These unique phenotypes provide further evidence that each SS isoform serves a role in starch biosynthesis that cannot be supplied by any of the other enzyme classes. Genetic data alone, however, cannot fully determine the specific physiological functions of any enzyme because the phenotypic effects on starch structure might not necessarily be direct. Such considerations are important in light of the findings that SSI, SSII, and SSIII from either wheat or maize are capable of assembling with each other into various multiple subunit complexes [31,32]. Thus, analysis of plants lacking multiple SSs may be informative regarding the comprehensive mechanisms by which the chain length distribution within amylopectin molecules is determined. This approach has been reported previously using antisense technology to simultaneously repress the levels of SSII and SSIII in potato tubers, and synergistic effects indicative of functional interactions between the two enzymes were observed [22,27]. Arabidopsis provides a tractable genetic system to further these studies owing to the ability to completely eliminate any SS class by null mutation and to couple these mutations in desired combinations by standard genetic manipulations. Also, studying SS functions in different organisms, and in a transient starch system in leaves compared to storage starches in tubers or other tissues, likely will reveal conserved biochemical functions that are significant in determination of amylopectin structure.

In this study analysis of SS functions in *Arabidopsis *leaves was extended by characterization of mutations in *AtSS2*, the gene coding for SSII, and by construction of a double mutant line that is completely deficient in both SSII and SSIII activity. Loss of SSII in *Arabidopsis *has effects very similar to those observed in other species. Elimination of SSII and SSIII together causes synergistic defects more severe than those resulting from mutation of only one of the two enzymes with regard to starch content and amylopectin structure. Thus, designation of SS function based on particular chain length ranges synthesized by each enzyme class is not likely to fully explain the conservation of multiple SSs. The data suggest that competition between different SS enzymes for binding to the ends of growing linear chains is an additional factor important for the determination of amylopectin structure.

## Results

### Identification of null mutations in the gene coding for SSII

**Figure 1:**
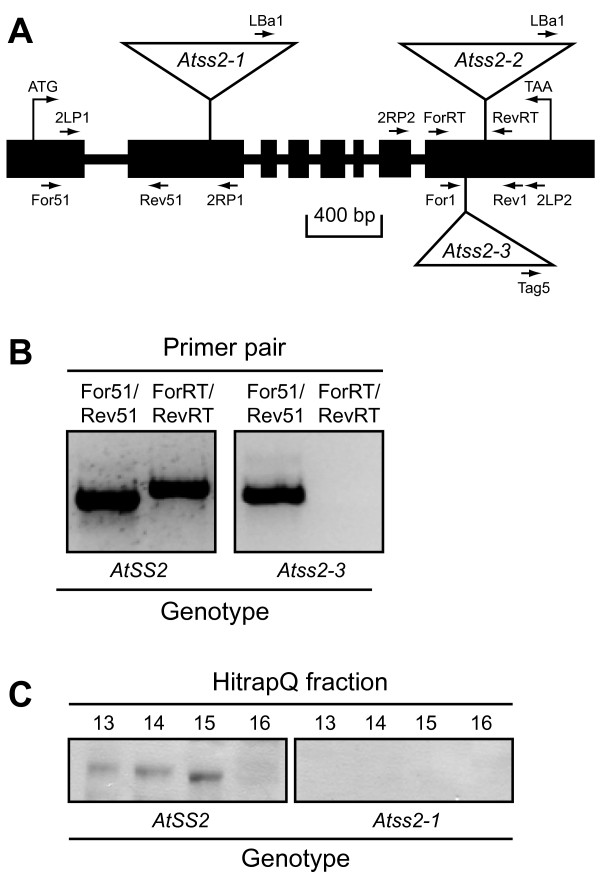
**Gene structure and allele verification**. **A**, *AtSS2 *gene map. The scaled linear map depicts the eight exons as black boxes and the seven introns as lines between the exons. The positions of the translational start and stop codons are noted. The locations of specific primer sequences used for PCR amplification are noted, as well as the locations of three T-DNA insertions in the gene. **B**, RT-PCR analysis of transcripts from the *Atss2-3 *mutant. Total RNA from leaves of WS wild type plants or the *Atss2-3 *mutant was amplified by RT-PCR using the indicated primer pairs. **C**, Immunoblot analysis. Total soluble proteins from crude leaf extracts were separated by anion exchange chromatography, then selected fractions were separated by SDS-PAGE and subjected to immunoblot analysis using anti-AtSSII antibody.

The *Arabidopsis *genomic locus At3g01180, hereafter referred to as *AtSS2*, codes for a protein highly similar in amino acid sequence to SSII proteins from rice, wheat, barley, pea, potato, and maize [21-26] that clearly falls into the conserved group of orthologous SSII genes distinct from any other SS [9-11]. Comparison of the genomic sequence to the corresponding cDNA sequence (Genbank accession number NM_110984) revealed that *AtSS2 *comprises eight exons and seven introns (Figure 1A). The 5'-untranslated region of the mRNA contains at least 168 nt upstream of the initiation codon. The computational programs ChloroP and TargetP [33,34] predict that the first 55 amino acids of the polypeptide coded for by *AtSS2 *are likely to function as a chloroplast targeting peptide, and the predicted molecular weight of the mature protein is approximately 81 kDa.

Three independent *Arabidopsis *lines with T-DNA insertion mutations in the *AtSS2 *locus were identified. Two T-DNA insertion mutant lines, Salk_065639 and Salk_102650, were obtained from the Salk collection [35], and a third T-DNA insertion line, EIB123, was obtained from the T-DNA mutant collection at INRA of Versailles, France [36-38]. Thus, all subsequent analyses were performed independently in two different *Arabidopsis *wild type backgrounds, Columbia for the Salk lines and WS for the INRA Versailles line. The mutations in these lines were designated as *Atss2-1*, *Atss2-2*, and *Atss2-3*, respectively.

PCR amplification of genomic DNA was used to identify homozygous mutant lines for each allele. Gene specific primer pairs flanking the approximate site of the T-DNA insertion identified the wild type allele, and one gene specific primer in combination with a T-DNA end primer revealed the mutant allele (Figure 1A) (primer pair sequences for genotype assignment are specified in Methods). The nucleotide sequences of the fragments amplified by one gene specific primer and a T-DNA end primer were determined in order to identify the exact insertion sites. These results showed that the T-DNA element in *Atss2-1 *is located in exon 2, and those in *Atss2-2 *and *Atss2-3 *are located at distinct sites in exon 8 (Figure 1A). Several successive generations of plants were genotyped by PCR analysis to confirm that each line was homozygous for its *Atss2 *allele.

RT-PCR analysis was used to confirm that the three T-DNA insertion in the *AtSS2 *locus effectively prevented expression of normal mRNA. For all three *Atss2 *alleles, primers flanking the known insertion site failed to generate any RT-PCR signal, whereas the same primer set generated a fragment of the expected size from wild type mRNA (Figure 1B and data not shown). Thus, normal *AtSS2 *transcripts are lacking in all three mutant lines. Some abnormal transcripts, however, do accumulate from the mutant genes. RT-PCR signal was detected when mRNA from an *Atss2-1 *plant was amplified using primers located in the downstream region of the gene (Figure 1A, primer pair 2LP2/2RP2). Similarly, amplification of an upstream region was detected from mRNA of an *Atss2-2 *plant (Figure 1A, primer pair 2LP1/2RP1) or an *Atss2-3 *plant (Figure 1A, primer pair For51/Rev51) (Figure 1B).

In the case of *Atss2-1*, immunoblot analysis was used to determine whether the mutation prevented accumulation of the protein product. Total soluble leaf extracts were first fractionated by anion exchange chromatography in order to partially purify SSII and thus facilitate its detection. Proteins in selected chromatography fractions were separated by SDS-PAGE and probed with a monoclonal antibody, referred to as αAtSSII, elicited using full-length recombinant *Arabidopsis *SSII as the antigen. Wild type extracts analyzed in this way revealed a signal at an apparent molecular mass of approximately 80 kDa (Figure 1C), which matches the predicted molecular mass for mature SSII after removal of the putative plastid targeting peptide. This band was not detected in the same chromatography fractions from *Atss2-1 *homozygous mutant leaf extracts (Figure 1C). These results, together with the RT-PCR data, indicate that *Atss2-1 *is a null allele in the sense that no detectable SSII protein is present in the homozygous mutant plants. *Atss2-2 *and *Atss2-3 *are also likely to be null alleles based on the demonstrated disruption of the mRNA structure.

### Construction of an *Atss2*, *Atss3 *double mutant and analysis of plant growth phenotypes

A double mutant was generated in order to examine for possible synergistic effects of simultaneous elimination of both SSII and SSIII. The particular alleles involved in the cross were *Atss2-1 *(Figure 1A) and *Atss3-1*, previously shown to be a null mutation [29]. Double homozygous mutant lines were identified at approximately the expected Mendelian ratio among the progeny of a double heterozygote, using PCR analysis to assign genotypes.

**Figure 2:**
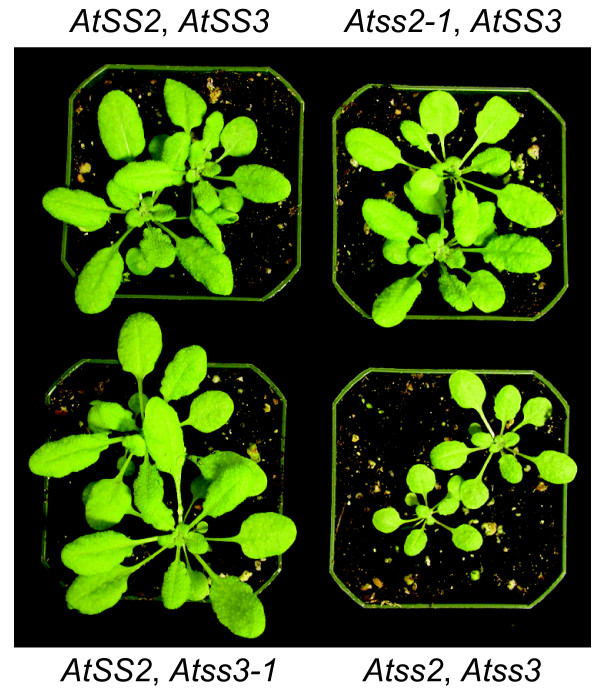
**Plant growth phenotype**. Plants of the indicated genotype were germinated in soil in a growth room under long day conditions (16 h light/8 h dark). Small plants were then transferred to pots and grown in a growth chamber under short day conditions (8 h light/16 h dark) for three weeks.

Single and double mutant plants were compared to wild type with regard to seed germination rate, plant growth rate, flowering time, and silique formation. When grown under constant light or under a 16 h light/8 h dark long day photoperiod (LD), no significant differences were noted between wild type, *Atss2 *single mutants, *Atss3 *single mutants, or the *Atss2*, *Atss3 *double mutant plants (data not shown). In contrast, when plants were grown under a 8 h light/16 h dark short day photopheriod (SD), a significantly reduced growth rate was observed uniformly for every *Atss2*, *Atss3 *double mutant tested as compared to wild type or either single mutant line (Figure 2). One double homozygous mutant line was characterized by RT-PCR to confirm that the normal mRNA for both of the genes was absent (data not shown), and this particular *Atss2*, *Atss3 *line was used in all further analyses.

### Effects of mutations on SS activity and other starch metabolizing enzymes

**Figure 3:**
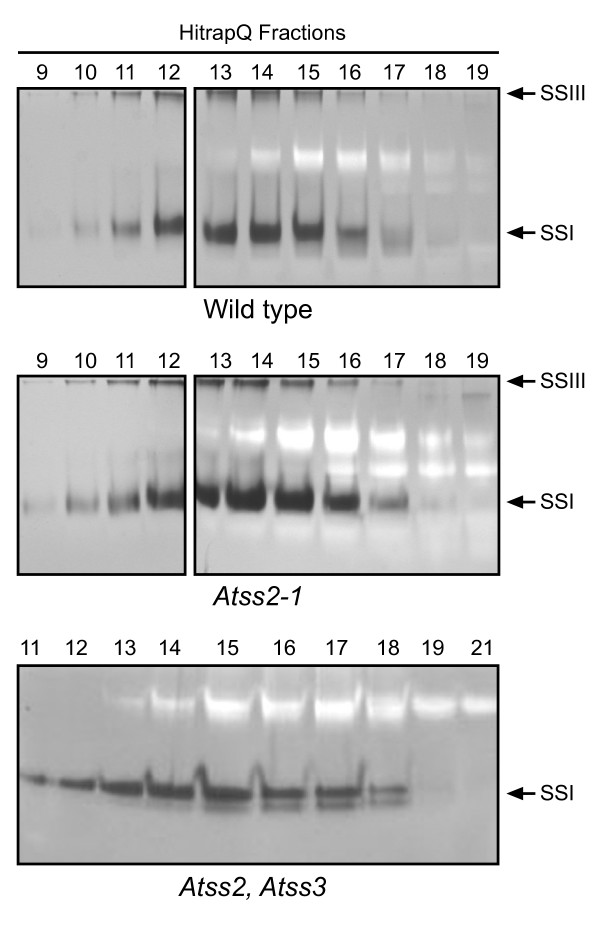
**SS activities observed by two dimensional zymogram**. Soluble leaf extracts from plants of the indicated genotype were separated by anion exchange chromatography followed by native PAGE in the presence of 0.3% glycogen. SS activity was then qualitatively assayed in place in the gel by addition of ADPGlc and subsequent staining with I_2_/KI solution.

Two-dimensional zymograms were used to determine the effects of the SSII single mutations and the SSII/SSIII double mutation on specific SS enzyme activities. Soluble leaf extracts were fractionated by anion exchange chromatography, and equal volumes of selected fractions were loaded onto non-denaturing PAGE gels containing 0.33% glycogen. After electrophoresis, the gels were incubated in a buffer containing ADPGlc and glycogen, and then stained with I_2_/KI solution. Gel zones that contain an active SS stain dark brown in these assays because the enzyme lengthens exterior glucan chains on the glycogen substrate. Two major SS activity bands were observed in wild type leaf extract (Figure 3). The more slowly migrating band was previously identified as SSIII [29]. The faster migrating band was identified as SSI based on its absence from *Atss1 *mutants [28]. The minor band visible below the major SSI band in the double mutant (Figure 3) also requires a functional *AtSS1 *gene (data not shown) and thus is identified as a form of that enzyme. The faint band visible in fractions 18 and 19 in the analysis of wild type and the *Atss2-1 *mutant in Figure 3 is not an SS activity because it appears even when ADPGlc is omitted from the incubation medium [29].

As expected SSIII was missing in the *Atss2-1*, *Atss3-1 *double mutant (Figure 3). No difference was observed in the SS activity pattern between the *Atss2-1 *line and wild type (Figure 3), and the wild type pattern was also observed for the *Atss2-2 *mutant (data not shown). The zymogram was also performed on total soluble leaf extract, i.e., without prior anion exchange fractionation, and again both *Atss2 *mutants exhibited the same pattern as wild type (data not shown). Therefore, SSII either is present in very low abundance in leaf extracts, or the zymogram assay is ineffective in detecting this particular SS form as compared to SSI or SSIII.

Total soluble SS activity in soluble leaf extracts was measured in the various mutant strains by an in vitro assay using ^14^C-ADPGlc as a substrate. Total SS activity was not significantly decreased in *Atss2 *mutant plants or the *Atss2*, *Atss3 *double mutant compared to wild type control lines (Table 1). The reason that eliminating two of the three major soluble SS classes does not affect total enzyme activity in the leaf extracts is not obvious, however, the explanation may involve increased SSI activity in the absence of the other SSs. This situation has been observed in other species, specifically maize and rice, where elevation of SSI activity in extracts lacking SSIII compared to wild type has been described previously [13,39,40].

**Table 1:** Starch metabolic enzyme activities in soluble leaf extracts

Enzyme	Activity by genotype			
	
	Wild type	*Atss2-1*	*Atss3-1*	*Atss2/Atss3*
SS^a^	212 ± 8.66 (4)	237 ± 8.74 (4)	265 ± 18.6 (4)	190 ± 8.78 (4)
SS	9.24 ± 0.80 (2)	9.19 ± 0.16 (2)	9.82 ± 0.07 (2)	9.85 ± 0.41 (2)
SBE^b^	50.3 ± 7 (1)	52.6 ± 9.9 (1)	45.8 ± 5.7 (1)	51.5 ± 10.0 (1)
ADPGPP	15.5 ± 3.8 (3)	19.9 ± 3.1 (3)	14.5 ± 2.4 (3)	14.5 ± 2.5 (3)
ADPGPP + 3PGA	35.3 ± 1.8 (3)	33.7 ± 4.0 (3)	22.1 ± 6.9 (3)	23.2 ± 13.0 (3)
α-amylase	32.6 ± 2.0 (3)	33.7 ± 3.4 (3)	35.5 ± 1.3 (3)	44.1 ± 3.7 (3)
β-amylase	191 ± 27 (3)	237 ± 46 (3)	259 ± 22 (3)	243 ± 30 (3)
Pullulanase-type DBE	8.40 ± 1.7 (3)	15.4 ± 1.8 (3)	2.06 ± 0.34 (3)^c^	3.90 ± 1.9 (3)
DE	25.6 ± 5.8 (3)	29.3 ± 3.0 (3)	30.4 ± 0.65 (3)	32.8 ± 1.8 (3)
Maltase	42.5 ± 4.7 (3)	37.6 ± 0.59 (3)	40.5 ± 4.9 (3)	38.5 ± 9.8 (3)
SP^b^	1.34 ± 0.20 (1)	2.86 ± 0.22 (1)	0.70 ± 0.35 (1)	0.97 ± 0.08 (1)

Unless otherwise noted, units are nmol product/min/mg protein. Values in parentheses indicate the number of independent biological replicates used to generate the mean and standard error values indicated.

Abbreviations are as follows: DE: disproportionating enzyme; SP, starch phosphorylase; ADPGPP: ADPGlc pyrophosphorylase; DBE: starch debranching enzyme; 3PGA: 3-phosphoglycerate.

^a ^SS activity was measured independently in two completely separate sets of assays. For the assays in this row only the units are nmol glucose incorporated/g FW/min.

^b ^The values for SP and BE activity were determined by triplicate measurements from a single plant extract.

^c ^Value is significantly different from wild type after Dunnet's adjustment for multiple comparisons. Unless so indicated, values are not significantly different from wild type.

**Figure 4:**
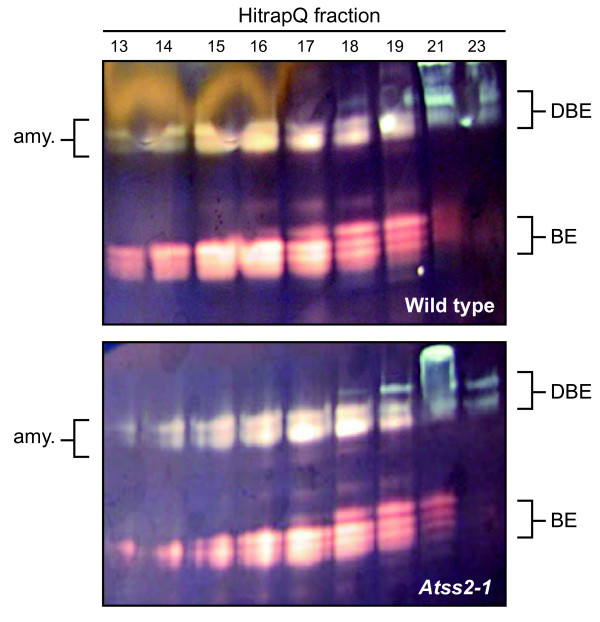
**Starch modifying activities observed by two dimensional zymogram**. Proteins in total soluble leaf extracts were separated by anion exchange chromatography and then by native PAGE. The proteins in these gels were transferred to a second polyacrylamide gel containing 0.3% potato starch. Gels were then stained with I_2_/KI solution.

Two dimensional starch zymogram analysis [41] was used to examine whether the mutations pleiotropically affect BE, DBE, or amylolytic activities. Anion exchange column fractions were separated by non-denaturing PAGE, and then the gels were electro-blotted to a second polyacrylamide gel impregnated with 0.3% starch. After incubation the gels were stained with I_2_/KI solution, so that colored bands above the dark background revealed the presence of starch modifying enzymes. No differences in the banding pattern were observed between the wild type and mutant lines for the isoamylase-type DBEs, BEs, or amylolytic activities detected in this assay (Figure 4 and data not shown).

Other starch metabolic enzymes were characterized by standard biochemical assays of total soluble leaf extracts, again to examine for major pleiotropic effects that could affect phenotype (Table 1). Only minor changes likely due to statistical variation were observed for total BE activity, ADPGPP (particularly in the absence of 3-PGA as an activator), α-amylase, β-amylase, disproportionating enzyme (DE), and maltase. Potentially significant changes were observed in the mutants with regard to pullulanase-type DBE and starch phosphorylase (SP), although the nature of these changes requires further investigation. Taken together the data indicate that the phenotypic effects of *Atss2 *mutation and *Atss2*, *Atss3 *double mutation likely result directly from the effects on SS as opposed to pleiotropic effects on other starch metabolic enzymes.

### Effects of mutations on glucan and sugar content

**Figure 5:**
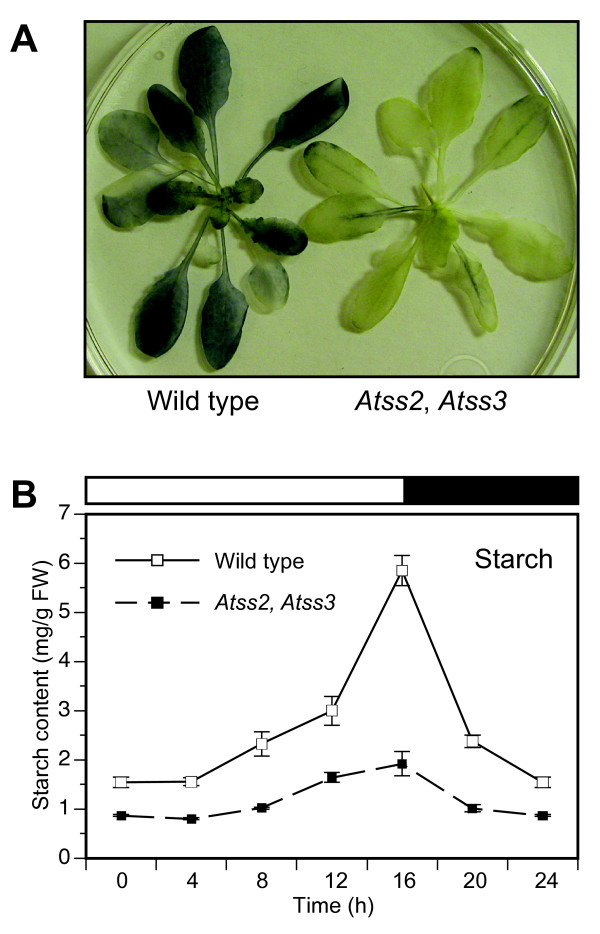
**Glucan content**. **A**, Iodine staining. Leaves were harvested at the end of the light phase from plants of the indicated genotype grown for several weeks in long day growth room conditions. These leaves were then decolorized, stained with I_2_/KI solution, and destained for 5 – 10 min in water. **B**, Starch quantification. Starch was isolated from leaves of plants grown under long day growth room conditions, harvested at specific times in the 16 h light/8 h dark diurnal cycle. Granules were collected by low-speed centrifugation and the glucan content in the pellet quantified. Each point is the average of leaves from six individual plants, and standard error is indicated.

Starch content was examined in leaves from the various mutants harvested throughout the diurnal cycle. As a first approximation, leaves collected at the end of the light phase of the LD cycle were decolorized by boiling in ethanol and then stained with I_2_/KI solution. In this assay there was no difference in the apparent starch content between wild type and *Atss2 *mutants, and the previously reported increase in staining intensity in the *Atss3 *mutants [29] was again observed (data not shown). In contrast to the single mutants, the *Atss2*, *Atss3 *double mutant appeared to contain very low amounts of starch as detected by staining of leaves with iodine (Figure 5A).

Starch content was quantified by chemical assay in leaves collected at the end of the light phase in plants grow in either the LD diurnal cycle, when all the mutant plants appear to grow at the same rate as wild type, or the SD cycle when the double mutant lacking both SSII and SSIII exhibits a clear growth defect. The results confirmed that *Atss2 *mutations do not cause a major change in leaf starch content and that in these growth conditions the *Atss3 *mutation conditions an apparent increase in starch content (Table 2), in agreement with previously published results [29]. The major decrease in starch content seen by leaf staining in the *Atss2, Atss3 *double mutant was also detected with clear statistical significance in the quantitative assay (Table 2) in either LD or SD growth conditions. In these two growth conditions the double mutant contained 28% or 53%, respectively, of the wild type starch content.

**Table 2:** Carbohydrate content

Genotype	Starch		WSP^a^		Sucrose	Glucose	Fructose
				
	LD	SD	LD	SD	SD	SD	SD
WT^b^	5.54 ± 0.35	4.39 ± 0.39	0.15 ± 0.03	0.04 ± 0.01	0.35 ± 0.09	0.15 ± 0.03	0.08 ± 0.02
*Atss2-1*^b^	5.22 ± 0.37	4.91 ± 0.20	0.14 ± 0.05	0.11 ± 0.02	0.33 ± 0.05	0.15 ± 0.04	0.06 ± 0.01
*Atss2-2*^b^	6.16 ± 0.30	ND	ND	ND	ND	ND	ND
*Atss3-1*^b^	6.77 ± 0.24	6.02 ± 0.11^c^	0.14 ± 0.04	0.10 ± 0.04	0.30 ± 0.09	0.22 ± 0.07	0.08 ± 0.01
*Atss2*, *Atss3*^a^	1.56 ± 0.07^d^	2.34 ± 0.29^d^	0.16 ± 0.01	0.19 ± 0.04^d^	0.36 ± 0.08	0.69 ± 0.10^d^	0.26 ± 0.04^d^
WT^c^	8.60 ± 0.54	ND	ND	ND	ND	ND	ND
*Atss2-3*^c^	7.00 ± 0.60	ND	ND	ND	ND	ND	ND

^a^WSP indicates water soluble glucan polysaccharide

^b^Columbia genetic background

^c^WS genetic background

^d^Values significantly different from wild type after adjustment for multiple comparisons. Any values not so noted were not significantly different from wild type.

Units are mg/g FW. The values shown are the mean and standard deviation from four independent leaf extractions, each of which was assayed in duplicate. Abbreviations are as follows: LD: 16 h light/8 h dark long day diurnal cycle; SD: 8 h light/16 h dark short day diurnal cycle; ND: not determined.

Starch content was also quantified at various times through a single LD diurnal cycle in the *Atss2*, *Atss3 *double mutant as compared to wild type. The reduced granular starch content of the double mutant was evident throughout the entire cycle (Figure 5B). The timing of starch synthesis was not affected in the *Atss2*, *Atss3 *line, as accumulation was noted at the end of the light phase. Starch content in the *Atss2-1 *single mutant was also quantified throughout the diurnal cycle. In this instance there were no major differences from the wild type values (data not shown).

The levels of water-soluble glucan polysaccharide (WSP) were quantified in order to examine whether a glycogen-like polymer or low-molecular weight maltooligosaccharides (MOS) might accumulate in the *Atss2*, *Atss3 *double mutant in place of crystalline starch granules. Statistically significant differences from wild type were not observed in LD conditions, however, in SD conditions this component was elevated in the double mutant approximately 4.5-fold compared to wild type (Table 2).

Simple sugar levels also were quantified in plants grown under SD and LD conditions. In SD conditions sucrose levels were not significantly different from wild type in either single mutant line or the *Atss2*, *Atss3 *double mutant (Table 2). Monosaccharide levels in SD-grown plants, specifically glucose and fructose, were elevated in the double mutant compared to the other strains (Table 2). The increase compared to wild type was approximately 4.5-fold for glucose and 3.4-fold for fructose, and these differences were statistically significant. In contrast to the SD conditions, LD-grown plants did not exhibit significant difference between the *Atss2, Atss3 *double mutant and wild type (data not shown). The reason that the double mutation has significant effects on soluble carbohdyrate content, i.e., WSP, glucose, and fructose, in SD conditions but not in LD conditions, is not obvious but may be related to the rate of starch accumulation in the different diurnal cycles.

### Effects of mutations on starch structure and composition

#### Granule morphology

**Figure 6:**
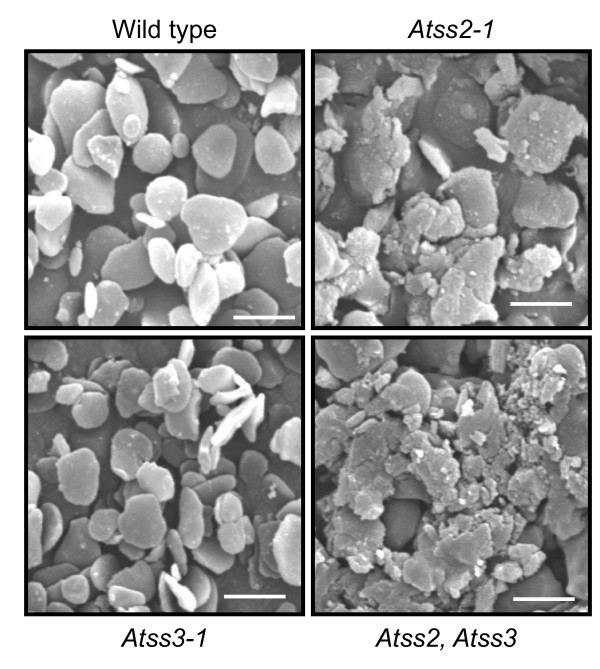
**Starch granule morphology**. Purified starch granules from plant leaves of different genotype were coated with gold particles, visualized by scanning electron microscopy, and then photographed. Bar = 2 μm.

The morphology of leaf starch granules isolated from wild type and the various mutant lines was examined by scanning electron microscopy (Figure 6). As previously reported, starch granules of the *Atss3-1 *mutant have normal morphology [29]. Granules from both *Atss2-1 *and *Atss2-2 *are distorted in shape and seemingly larger than those of the wild type control (Figure 6 and data not shown). Starch granules in the *Atss2*, *Atss3 *double mutant are also distorted in shape, similar to the *Atss2 *single mutants, and the phenotype appears to be more exaggerated in the double mutant line.

#### Amylose content and amylose/amylopectin ratio

**Figure 7:**
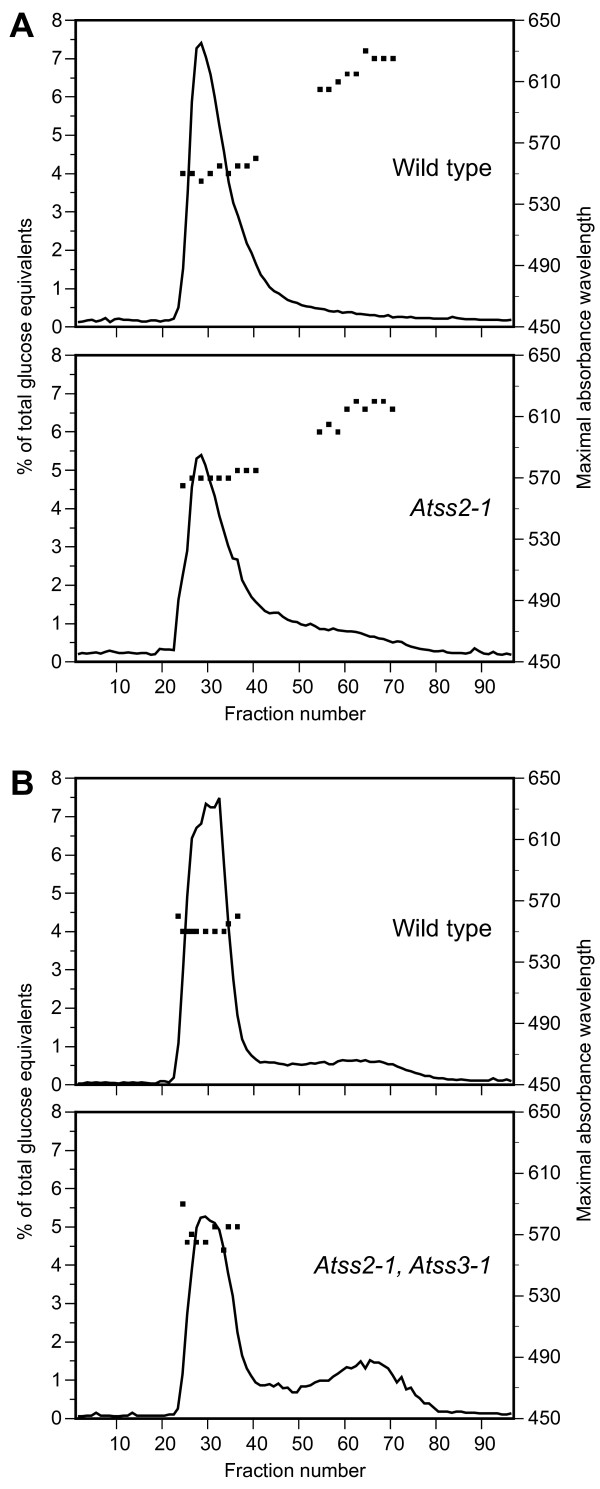
**Glucan separation by gel permeation chromatography**. **A**, Wild type and *Atss2-1*. Purified granules from plants of the indicated genotypes were dissolved by boiling in DMSO, and the polymers present were separated by GPC on Sepaharose CL-2B on a column measuring 1.8 cm i.d. × 1 m height. Glucan polymer in each fraction was enzymatically quantified, and samples of each fraction were stained with I_2_/KI solution. Visible absorbance spectra were recorded and the maximal absorbance wavelength is noted. **B**, Wild type and *Atss2*, *Atss3*. Analysis was as in panel A, except that the column size was 1.2 cm i.d. × 50 cm height.

The relative amounts of amylose and amylopectin in starch granules were compared between wild type and the three mutant lines after separating starch polymers by gel permeation chromatography (GPC). The apparent amylose peak from the *Atss2 *mutant lines exhibited a maximal absorbance wavelength when complexes with iodine (λ_max_) of approximately 610 nm, closely matching the wild type value (Figure 7). Thus, the slower-eluting glucan present in the mutants is identified as amylose, as opposed to a modified amylopectin of anomalously low molecular mass. The single measurement of the *Atss3-1 *mutant showed no obvious difference in amylose:amylopectin ratio compared to wild type (Table 3), confirming the previously published result [29]. All three *Atss2 *mutations conditioned an increase in the apparent amylose content. In the WS background, the wild type starch contained 25% amylose and the *Atss2-3 *mutant contained 43% amylose, and this difference was shown to be statistically significant (Table 3). Both *Atss2 *mutant alleles in the Columbia background also exhibited increased amylose content compared to wild type (Figure 7A, Table 3). These results are similar to the reported effect of SSII deficiency on starch composition in other species [21-26]. In the single analysis of the *Atss2, Atss3 *double mutant starch an even greater increase in amylose content was observed (Figure 7A, Table 3).

**Table 3:** Leaf starch properties

Genotype	Amylose content		Amylopectin λ_max_	Phosphate
			
	%	(mg/g FW)		
Wild type^a^	21.6 ± 1.4 (5)	1.20	545 nm	4.35 ± 0.43 (2)
*Atss3-1*^a^	20.7	1.40	555 nm	10.7 ± 0.03 (2)
*Atss2-1*^a^	33.7	1.75	565 nm	3.01 ± 0.24 (2)
*Atss2-2*^a^	34.2	2.11	565 nm	NA
*Atss2*,*Atss3*^a^	40.1	0.63	575 nm	NA
Wild type^b^	25.2 ± 1.8 (5)	2.17	549 nm	NA
*Atss2-3*^b^	42.7 ± 6.7 (5)^c^	2.99	555 nm	NA

^a^Columbia genetic background

^b^WS genetic background

^c^Value significantly different from wild type as indicated by P-value < 0.05.

Starch was analyzed from leaves harvested at the end of the light period of the LD diurnal cycle. GPC fractions 42 and lower were designated as amylopectin and fractions 43 and higher were designated as amylose (Figure 7). Parentheses indicate the number of independent biological replicate assays used to generate the mean and standard deviation. The *Atss2-1 *and *Atss2-2 *mutant lines were analyzed only once, however, these assays are independent biological replicates for the condition in which SSII is absent. Amylose content was normalized to leaf fresh weight by multiplying the normalized total starch amount from Table 3 by the amylose percentage determined for each genotype. Phosphate content units are nmol phosphoryl groups/mg starch. "NA" indicates data not available. Data for *Atss3-1 *were previously published [29] in a study using identical analytical methodology.

In addition to the increased ratio of amylose to amylopectin, increased total accumulation of amylose was observed in all three SSII single mutant lines. Normalizing the amount of amylose to the mass of leaf fresh weight revealed increases between 38% and 76% (Table 3). This effect was not observed in the *Atss2, Atss3 *double mutant, presumably because of the low total starch content in that line.

#### Phosphate content

The phosphate content of starch is known to be influenced by SS mutations, either positively or negatively depending on the affected isoform [27,29,42]. To extend this analysis the phosphoryl group frequency in *Atss2-1 *mutant starch was quantified, with the result that phosphate content was decreased by approximately 30% compared to wild type (Table 3). This is in contrast to *Atss3 *mutations, which are known to result in significantly increased phosphate content [29]. The phosphate content could not be determined in the *Atss2, Atss3 *double mutant owing to sample limitation.

#### Amylopectin structure

Absorption spectra of glucan-iodine complexes indicated distinct amylopectin structures in the mutants as compared to wild type. In the Columbia background the λ_max _for both *Atss2-1 *and *Atss2-2 *mutants was 565 nm, whereas wild type amylopectin had a λ_max _value of 545 nm (Figure 7, Table 3). Elevated λ_max _for the *Atss2*-*3 *mutant compared to wild type in the WS background was also observed (Table 3). The λ_max _value for amylopectin from the *Atss2*, *Atss3 *double mutant was further elevated to 570 nm (Figure 7B, Table 3), indicating a structure different from either wild type or the *Atss2 *single mutants.

**Figure 8:**
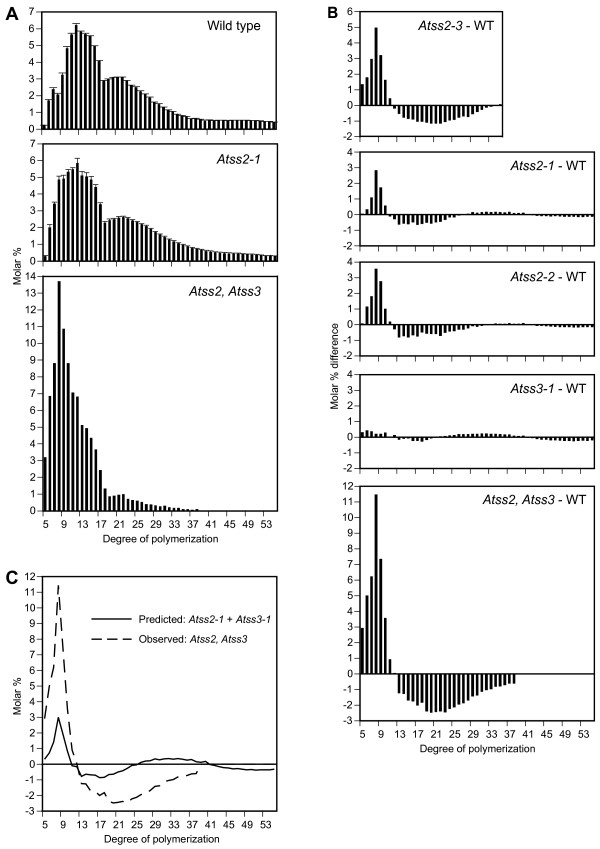
**Amylopectin chain length distribution**. **A**, Chain length distributions. The population distribution of chains of a given DP was normalized to the total number of chains in the DP range of 5–55. For wild type and *Atss2*-*1 *the values shown are the mean from at least three independent biological replicates, with error bars indicating the standard error. Starch from the *Atss2*, *Atss3 *double mutant was analyzed only once owing to sample limitation. **B**, Difference plot comparison of chain length distributions. For each given DP, the distribution mean value for wild type was subtracted from that of the indicated mutant strain. The chain length distribution means used to generate the difference plots for the *Atss2-2 *and *Atss2-3 *lines are taken from data not shown. Appropriate wild type controls were used, i.e., Columbia for the *Atss2-1 *and *Atss2-2 *mutants, and WS for the *Atss2-3 *mutant. For comparison, the difference plot for the *Atss3-1 *line is shown from previously published results [29]. **C**, Pleiotropic effect plot. The predicted sum of the *Atss2-1 *and *Atss3-1 *effects in single mutants (solid line) is compared to the observed result for the double mutant (dotted line).

The glucan chain length distribution in total leaf starch was determined for wild type and the mutant lines using fluorophore-assisted carbohydrate electrophoresis (FACE) [43,44]. The frequency of each individual chain length was calculated as a percentage of the total chains analyzed (Figure 8A). To enable comparison between genotypes, the normalized wild type distribution value for each chain length was subtracted from the equivalent value for a particular mutant (Figure 8B). Positive values indicate enrichment of that chain length in the mutant relative to wild type, whereas negative values indicate reduced frequency of such chains.

The difference plots for *Atss2-1 *and *Atss2-2 *are very similar, showing a significant enrichment in chains from DP5 to DP10 and depletion in chains from DP12 to DP28 (Figure 8B). A qualitatively similar result was observed for the *Atss2-3 *mutant analyzed in the WS genetic background, with the distinction that the degree of difference was larger compared to the Columbia lines (Figure 8B). In a previous report the *Atss3-1 *and *Atss3-2 *mutations were shown to have very small changes in these chain length ranges [29] (Figure 8B). The double mutant shows enrichment and depletion in the same chain length regions as those affected by the *Atss2 *single mutants, but the degree of the change was extreme (Figure 8B). In the most extreme case, DP8 chains represented 2.2% of the normalized total in wild type or the *Atss3-1 *mutant, 5.6% in either of the *Atss2 *mutants, and approximately 14% in the *Atss2*, *Atss3 *double mutant (Figure 8A and data not shown). Thus, the *Atss2*, *Atss3 *double mutant profile was quantitatively distinct from the sum of the patterns of the *Atss2 *and *Atss3 *single mutants (Figure 8C).

## Discussion

### *Atss2 single *mutation

The phenotype described here for amylopectin structure and amylose content can be definitively assigned as a result of the *Atss2 *mutations, as opposed to any other unidentified mutation(s) present in the T-DNA insertion lines. Three independent alleles of the *AtSS2 *locus were analyzed for phenotypic effects, and in all instances essentially identical results were obtained regarding starch structure and amylose content. Each T-DNA insertion allele originated in a distinct line as an independent event, so the probability is negligible that any unidentified second mutation in the genetic background could be the causative agent of the phenotype. Furthermore, major effects of the *Atss2 *mutations on other starch metabolic enzymes were not detected, so that pleiotropic effects are not a likely explanation for the changes in starch structure.

The effects of eliminating SSII on amylopectin structure in *Arabidopsis *leaves closely resembled the phenotype caused by SSII deficiency in other plant species including maize, barley, wheat, potato, rice, and pea [18,21-27]. In all these species SSII mutations cause altered amylopectin structure such that the frequency of linear glucan chains of DP6 to DP10 is significantly increased while the abundance of DP12 to DP28 chains is decreased. Like most of the other plant species, *Arabidopsis *granules exhibit distorted morphology when SSII is inactive.

From these observations it is clear that the physiological function of SSII is highly conserved among plants. The current study indicates that this function also applies to transient starch in leaves, in addition to storage starch in the other species analyzed previously. This conservation applies regardless of the abundance of SSII activity in the soluble phase of each tissue examined. For example, in pea embryos SSII represents up to 60–70% of the total soluble SSII activity [14], in pea leaves SSII is a minor but detectable isoform and SSIII accounts for the major portion of the activity [45], and in the current study SSII was undetectable in soluble extracts of *Arabidopsis *leaves. A possible explanation for this apparent discrepancy may be that the portion of SSII that is present within granules is the determining factor in amylopectin structure, as opposed to that in the soluble fraction. This parameter has not been tested in *Arabidopsis *leaves, however, in all storage starches examined SSII has been observed to partition to some extent within the insoluble granules [14,16,18,21,23,26,46,47]. Another possible explanation is that the in vitro measurement of SSII activity does not accurately reflect the enzyme's activity in vivo, owing to an unidentified effect of the assay conditions employed.

Analysis of leaf starch offered an advantage in observing the effects of SSII on total starch levels, because during the diurnal cycle the starch content is reduced to near zero at the end of the dark phase. Thus, the starch that accumulates during the light phase is synthesized nearly entirely over the previous 16 h in the light regime used in this study. This is in contrast to storage starch that accumulates over a period of weeks. The observed result is that SSII mutation did not cause a major change in the starch levels at the end of the day, indicating that despite the significant change both in amylopectin structure and amylopectin/amylose ratio the overall rate of starch accumulation is not decreased by loss of SSII.

An interesting feature of starch biosynthesis in all three *Atss2 *mutants is that the amount of amylose produced in leaves relative to the total tissue fresh weight is increased. The fact that the percentage of amylose in starch granules rises in the absence of SSII was known from previous studies in other species. This effect on relative amylose content could result from decreased amylopectin directly caused by the SS defect. The current study, however, demonstrates that the absolute amount of amylose is increased in the mutants over that found in wild type leaves. Presumably the activity of GBSS, the isoform responsible for amylose production, is increased in *Atss2 *mutant granules compared to wild type.

Increased abundance of GBSS does not appear to be the explanation for the elevated total amylose content, because approximately equal amounts of this enzyme were observed by immunoblot analysis in wild type and *Atss2-3 *mutant granules (CD and NS, unpublished results). Possible explanations for the increased amylose content are a direct regulation of GBSS by SSII, or that the altered amylopectin structure resulting from loss of SSII affects the GBSS activity. The latter explanation could occur through an allosteric regulation of GBSS by the glucan environment in which it is located [48]. Alternatively, it has been proposed that GBSS directly uses amylopectin as a precursor to the synthesis of amylose [49]. According to this hypothesis the structurally altered amylopectin of the *Atss2 *mutants may provide a more effective substrate for GBSS, allowing greater levels of amylose production. Yet another possible explanation for the increased total amylose content is that elevated MOS levels stimulate GBSS activity by acting as a primer, as has been demonstrated in vitro [50]. This explanation is unlikely to apply, however, because WSP levels were not elevated in any of the mutants grown in LD conditions (Table 2) yet the total amylose increase was clearly evident (Table 3).

### *Atss2, Atss3 *double mutation

Combining mutations that eliminate SSII or SSIII in a single line provided a test of whether or not those two enzymes act independently in starch biosynthesis. Previous analysis of *Atss3 *mutants showed no change in amylose content or granule morphology, and only minor changes in amylopectin chain length distribution on a much smaller scale than were observed here for *Atss2 *mutation [29]. Accordingly, if SSII and SSIII act independently then the expected amylopectin structure in the double mutant would be the nearly same as that of the *Atss2 *single mutants. To the contrary, the starch phenotype of the double mutant was far different than that of either *Atss2 *or *Atss3 *single mutants. The most likely explanation for these results is that SSII and SSIII are capable of executing the same or similar functions in the determination of amylopectin structure.

The chain length range affected in the double mutant is the same as in the *Atss2 *single mutants, however, the magnitude of the change is far greater. In fact, the glucan structure of the *Atss2, Atss3 *line is highly abnormal and resembles glycogen more than it does amylopectin, even though the mutant polymer is still present in water-insoluble granules. Synergism is also evident in the starch content, which is reduced in the double mutant by more than 70% at the end of the day. This compares to no reduction in content owing to the SSII deficiency, and an increase in the starch content conditioned by SSIII mutation in the growth environment used in this study.

The current study confirms the synergistic effects of simultaneous reduction in SSII and SSIII observed previously in antisense potato plants [22,27], and in addition reveals several distinctions between the leaf and tuber systems. First, the total amounts of starch produced were not affected in SSII/SSIII antisense tubers [22,27], whereas the *Arabidopsis *mutant lacking both enzymes clearly exhibited a starch deficiency. This effect is likely due to the nature of the transient system in which starch present at the end of the light phase accrues during the course of single day, compared to tubers where starch accumulates steadily over a much longer period of time. Second, more severe effects on chain length distribution were detected in *Arabidopsis *double mutant compared to the potato antisense plants. Most notably, the extreme magnitude of the synergistic effects of simultaneous loss of SSII and SSIII on chains of DP6 to DP10 is most clearly observed in the current study. The technique applied for measurement of chain length frequency in one of the potato antisense studies prevented accurate quantification of the synergistic effects [27]. In the other antisense study in potato tubers synergism was detected regarding the DP range of the chains that were affected, however the change in relative abundance of any particular length chain was the same in the single and double antisense lines [22]. This result is different than the synergistic effects observed in *Arabidopsis *leaves. A possible explanation for the differences is that in Arabidopsis the insertion mutations completely eliminated each enzyme activity, whereas in potato the SS activities were reduced but not necessarily lost entirely. Alternatively, although the individual roles of each SS appear to be conserved in plants, the nature of the interactions in these two comprehensive biosynthetic systems may vary. These observations point out the utility of the *Arabidopsis *genetic system.

The observed changes in starch metabolism in the *Atss2, Atss3 *double mutant occurred despite the fact total SS activity in soluble leaf extracts was not significantly reduced compared to wild type (Tables 1 and 2). This observation is unexpected in light of the fact that SSI activity appears to account for only approximately 50% of the total, as determined by analysis of *Atss1 *mutants [28]. However, neither *Atss2 *nor *Atss3 *mutations alone diminish total SS activity (Tables 1 and 2) [29]. This apparent paradox may be explained by the proposal that SSIII serves as a negative regulator of SSI [29], potentially through interactions in the same multi-subunit complex [31], so that SSI activity could be elevated compared to wild type in the double mutant. Similar observations were made in maize endosperm extracts in which mutations affecting SSIII caused increased total SS activity as opposed to the expected reduction [13,39], and nearly all of the elevated activity could be attributed to SSI [13]. A similar relationship also was demonstrated in rice endosperm, in which SSI activity was shown to be elevated 1.3 to 1.7-fold as the result of a mutation affecting SSIIIa [40].

These considerations emphasize the point that total SS activity is not entirely responsible for amylopectin structure determination, and that the relative abundance of each conserved class of SS enzyme likely is an important factor. Similarly, total soluble activity is not solely responsible for determining starch content, because the *Atss2, Atss3 *double mutant is deficient in starch even though the remaining SS classes are able to provide a high level of activity. Either SSII or SSIII, acting in concert with SSI, are able to support the wild type level of starch synthesis. A possible explanation of this fact is that SSI may be unable to extend chains to a length suitable for BE action, whereas either SSII or SSIII can produce suitable BE substrate chains.

### Functional interaction among SS classes

The effects of single and double mutations in *Arabidopsis *genes raise the idea that normal amylopectin chain length distribution is determined in large part by competition between different SSs for binding to any glucan chain substrate along with the inherent ability of each SS class to catalyze elongation of chains of particular lengths. The latter point is illustrated by the extreme deficiency of chains of DP12 to DP25 in the *Atss2, Atss3 *double mutant. In this instance the major soluble SS remaining is SSI. Thus, this enzyme appears to be unable to elongate glucan chains much beyond DP10, which is consistent with the known biochemical properties of maize SSI measured in vitro [51].

Competition between SSI and SSII or SSIII for binding to each glucan chain is suggested from the phenotype caused by the *Atss1 *mutation [28] or the analogous mutation in rice [52]. Amylopectin from these mutants exhibits increased frequency of chains in the length region of DP12 to DP28 and a relative deficiency in short chains of DP6 to DP10. In the absence of SSI, access of SSII or SSIII to substrate chains may be increased owing to reduced competition with SSI. If the catalytic efficiency of SSII or SSIII relative to chain length is extended compared to SSI, then the population of DP13 to DP25 chains would rise compared to the wild type condition in which SSI to some extent prevents SSII or SSIII action. In this view, SSI may bind to the shorter chains of approximately DP10 but have a low probability of catalytic activity, thus acting as a negative regulator of SSII or SSIII by means of steric interference. The balance between short chains of DP6 to DP10 and intermediate chains of DP12 to DP28 would be determined by the relative abundance of the three enzymes, their affinities for glucan chains as a function of DP, and their catalytic efficiencies after binding to substrate, again as a function of DP. The proposal that different SS classes would compete with each other for binding to potential substrate chains is consistent with the observation that SSII from pea embryos is a distributive enzyme that dissociates from the glucan substrate after each glucose unit addition [48,50]. Thus SSII and SSIII might compete for binding to potential glucan substrates during each cycle of chain elongation.

In order to explain the chain length profile of the *Atss2, Atss3 *double mutant, SSIII is proposed to partially overlap with SSII with regard to the DP region suitable for catalysis, but not with that of SSI. In the absence of SSII, SSIII would still be available to produce chains in the intermediate length region, and to compete with SSI for binding to potential substrates. SSIII is proposed to compete with SSI less effectively than does SSII, in order to explain the reduction in intermediate length chains of DP12 to DP20 in the *Atss2 *mutants. The fact that the *Atss3 *mutation alone has only a minor effect on amylopectin chains shorter than DP40 could be explained by a competitive advantage of SSII over SSIII for binding to short glucan chains. Accordingly, SSIII would not be involved to a great extent in intermediate chain length production in the wild type condition, but could provide that function in the absence of SSII.

## Conclusion

The activity of SSII is required in *Arabidopsis *for production of the normal frequency of amylopectin chains of DP12 to DP25, and thus the protein exhibits the same function that is conserved in numerous other plant species. None of the other SS classes can completely compensate for loss of SSII, however, SSIII is able to partially function in production of DP12 to DP25 chains. SSIII is not required for the normal population of these chains. These observations can be explained by competition between SSII and SSIII for binding to the non-reducing end of each potential substrate chain. Finally, loss of SSII affects the activity of GBSS such that the total amount of amylose in leaves is increased.

## Methods

### Plant materials and growth conditions

Wild type *Arabidopsis thaliana *of the ecotype Columbia (Col-0), and *Atss2-1 *or *Atss2-2 *mutant lines in this background, were sown in Sunshine Soil mix. Sown seeds were incubated at 4°C for 2–3 days then grown either in a growth room under a 16-h light/8-h dark photoperiod at 21°C and 60% relative humidity, or in a growth chamber under a 8-h light/16-h dark photoperiod and the same temperature and relative humidity. These growth facilities are located at Iowa State University in Ames, IA. Wild type *Arabidopsis *of the WS background, and the congenic *Atss2-3 *mutant line, were grown similarly. The growth facilities used for all lines in the WS background were located at the University of Science and Technology of Lille (USTL), Villeneuve d'Ascq, France. All subsequent analyses of the Columbia and WS lines were performed independently, the former at Iowa State University and the latter at USTL. Appropriate wild type control lines were used in all instances, i.e., Columbia for the *Atss2-1 *and *Atss2-2 *mutants, and WS for the *Atss2-3 *mutant.

### Identification of mutant alleles by PCR, and generation of mutant lines

Standard methods were used for isolation of genomic DNA from leaf tissue, and PCR amplification. PCR primers used to identify the mutant alleles were derived from the T-DNA left border (**LBa1**, TGG TTC ACG TAG TGG GCC ATC G) and from the *AtSS2 *gene sequence (**2RP1**, GCT ACC AAT ATC ACA TTC ATG AC; **2LP1**, CTT ACC ATG ATT TGC CTT CTG; **2LP2**, CCT CTT CTC TGA AGC CCT TCC C; **2RP2**, AGT GGT GGA AAA TTA GGG GCG) (Figure 1A). Nucleotide sequence analysis of PCR products generated by the LBa1/2RP1 primer pair or the LBa1/2LP2 primer pair revealed the genomic location of the T-DNA insertion generating *Atss2-1 *or *Atss2-2*, respectively (Figure 1A). The primer sets used for identification of *Atss2-3 *in the WS genetic background were as follows: **Rev1**, TGG TTC CAT AGT TCA TTG CGT AAA; **For1**, GAA GGA GGT TGG GGT CTG C; **Tag5**, CTA CAA ATT GCC TTT TCT TAT CGA C (Figure 1A).

The *Atss2*,*Atss3 *double mutant was generated by crossing homozygous *Atss2-1 *and *Atss3-1 *homozygous single mutant lines, then allowing the double heterozygous F1 plants to self pollinate. Genomic DNA from segregants was screened by PCR to reveal the presence of each insertion allele. The mutant allele *Atss2-1 *was revealed by amplification with the LBa1/2RP1 primer pair, and the wild type allele *AtSS2 *was identified by the 2RP1/2LP1 primer pair. Primer combinations for identification of *Atss3-1 *and the wild type *AtSS3 *allele were LBa1/SS3-TU1 and SS3-TU1/SS3-TL1, respectively [29]. One double homozygous line identified in the F2 generation was propagated for several more generations, and PCR genotyping consistently revealed all individuals in the lineage to be double homozygous mutants (data not shown).

### RT-PCR

Total RNA was isolated from approximately 100 mg of fresh leaf tissue from leaves harvested approximately 20 days after germination using the RNeasy Plant Mini Kit (Qiagen). Total RNA was treated with RNase-free DNase (Promega) (1 U, 30 min, 37°C) to remove any contaminating genomic DNA in the samples. A commercial enzyme kit (Invitrogen Superscript III) was used to synthesize cDNA. The sequences of the primers used to amplify the *AtSS2 *mRNA are specified in the previous section, or were as follows: **For51**, GCT GAG GCA TTC CCG TGT TTC T; **Rev51**, TGC GGT TCT TCA AGG ATT CAG TA; **ForRT**, GGG GAC CGG TAG ATG ATT TC, and **RevRT**, CGG TCG CCC TGT GCC TAA C. The primers used to amplify the *AtSS3 *mRNA were described previously [29], except for 3-EU1 located near the *AtSS3 *initiation codon (GGG GAC AAG TTT GTA CAA AAA AGC AGG CTT CCT GGT GCC ACG CGG TTC CGG AAG TGC TCA GAA AAG AAC).

### Expression of recombinant SSII in *E. coli *and monoclonal antibody production

Cloned, full length SSII cDNA was obtained from Riken (resource number: pda04163). The coding region of the SSII cDNA, minus the 120 nucleotide sequence at the 5' end coding for a predicted transit peptide, was cloned into pDONR201 using Gateway cloning technology (Invitrogen) to generate plasmid pESS2. The native stop codon of the SSII cDNA was included in the cloned region. The SSII cDNA sequence was then transferred by in vivo recombination to the expression vector pDEST15 to create expression plasmid pDSS2. In pDSS2, the SSII coding region is fused at its 5' end to a sequence coding for glutathione-S-transferase (GST). SSII was expressed from pDSS2 in *E. coli *BL-21 AI cells (Invitrogen). The fusion gene was induced by addition of 0.2% arabinose and induced cells were grown at room temperature for approximately 6 h. The SSII fusion protein was purified from *E. coli *lysates by binding to glutathione-agarose (Sigma) on a GST affinity column. Monoclonal antibody against this full length SSII-GST recombinant protein was generated as described previously [29]. The hybridoma culture fluid containing SSII antibody (αAtSSII) was used undiluted for immunoblot analysis.

### Partial purification of leaf proteins and enzyme activity measurements

Leaf extracts were prepared and fractionated by anion exchange on HiTrapQ (Pharmacia) as described previously [29]. Proteins from selected fractions were analyzed by immunoblot using α AtSSII monoclonal antibody. Fractions or whole leaf extracts were also analyzed by starch synthase zymogram [29] or by native starch zymogram [43], as described previously.

Total soluble starch synthase activity in leaf extracts was assayed essentially as described previously [13]. Fresh leaves (200 mg) were ground into fine powder in liquid nitrogen and suspended in 400 μL Extraction Buffer (50 mM Tris-acetate, pH 7.5, 10 mM DTT). After centrifugation to remove insoluble materials, protein concentration was determined by the Bradford method, and the starch synthase activity assay was then conducted with different quantities of protein. Control experiments demonstrated that the amount of ^14^C incorporated into methanol-precipitable product is linear with respect to the amount of protein in the assay (data not shown). Enzyme assays for BE, ADPGPP, α-amylase, β-amylase, DE, maltase, pullulanase-type DBE, and SP were performed as described previously [28,53].

### Analysis of starch quantity and structure

The methods used for analysis of starch content in leaves, amylopectin chain length distribution, granule morphology, starch phosphate content, and separation of amylose and amylopectin by GPC on Sepharose CL-2B have all been described previously [29,43]. Two different sizes of Sepharose CL-2B column were used, either 1.2 cm i.d. × 50 cm height, or 1.8 cm i.d. × 1 m height. The smaller column was eluted at a flow rate of 0.26 mL/min and 1.5 mL fractions were collected. The larger column was eluted at a flow rate of 0.5 mL/min and 3 mL fractions were collected. The starch granule glucans separated by GPC were characterized by staining the column fractions with 1/2 volume of I_2_/KI solution, obtaining a visible spectrum from 400 nm to 700 nm and then recording the λ_max _value. Glucan concentration in each fraction was quantified by determination of glucose units following complete hydrolysis with amyloglucosidase, using a commercial assay kit (Catalogue No. 10 207 748 035; R-Biopharm AG, Darmstadt, Germany)

In order to measure both WSP and insoluble glucans, i.e., starch, in the same leaf extracts, the ground leaf material suspended in 1 ml of water was centrifuged at full speed in a microfuge for 10 min, and the supernatant and pellet were both collected. The pellet was washed twice with 1 ml of 80% ethanol, and then suspended in 1 ml of distilled water. Glucan present in the soluble and granule fractions was enzymatically quantified as described in the previous paragraph. To quantify sucrose, D-fructose, and D-glucose, the pooled aqueous supernatant from six plants were quantified in triplicate with a Sucrose/D-Glucose/D-Fructose assay kit (Catalogue No. 10 716 260 035; R-Biopharm AG, Darmstadt, Germany).

### Statistical analyses

To evaluate the effects of SSII and/or SSIII mutations on starch and soluble carbohydrate content, and other starch metabolic enzymes compared to wild type, one-way ANOVA and two-sample t-tests were used for the comparisons. Since most of the experiments involved comparing single and/or double mutants to the common wild type control with multiple endpoints, Dunnett's and Bonferroni methods were used to adjust for multiple comparisons. Only experiments that had more than two independent replicates were considered for statistical analysis.

## Authors' contributions

XZ performed genetic manipulations, phenotypic determinations, and biochemical analyses involving the *Atss2-1 *and *Atss2-2 *single mutant lines and the *Atss2-1*, *Atss3-1 *double mutant, and helped to draft the manuscript. DD performed the genetic and biochemical analyses of the *Atss2-3 *mutant line. NS carried out biochemical assays of starch metabolic enzymes shown in Table 1. MGJ, CD, and AMM conceived the study and participated in its design and coordination, and helped to draft the manuscript. All authors read and approved the manuscript prior to submission.
